# Annotations

**Published:** 1889-08-31

**Authors:** 


### Annotations.

**Doctors and Paupers.**

Those persons who think that paupers are made in thousands by the wholesale flight of the working population to the out-patient department of the hospitals have secured an energetic leader in Dr. R. Rentoul, of Liverpool. Dr. Rentoul is spending time and strength in abundance in the cause of truth and publicity, and either he or somebody else must also be spending a good deal of money. That the facts brought to light by Dr. Rentoul at the meeting of the British Medical Association the week before last are startling, nobody who has mental capacity enough to understand them will for a moment deny. Unfortunately, in this world many people have a powerful influence upon practical affairs who have neither the capacity nor the wish to understand facts. So long as they themselves are prosperous and honoured, they cannot comprehend why all the rest of the world should not be perfectly satisfied. Here, for example, are two facts, which, when contrasted with one another, cannot fail to excite in every intelligent and conscientious Englishman a deep sense of shame. In England one person in every thirty-four is a pauper. That is not at all discreditable to the working people. But one person in every six receives charitable medical relief. That is a disgrace to at least three in every six. There are certain things which may be called prime necessaries of life. They stand in the first rank of indispensables. The most fundamental of them all is food. Will anyone for a moment contend that medical treatment is as prime a necessary as food? Yet whilst only one in thirty-four receives food for nothing, one in six receives medical treatment for nothing. Now, we may surely all make an effort for once to look at this fact, not as doctors, but as Englishmen. Let every man, both prosperous and unprosperous, both of *laissez faire* and of reforming proclivities, ask himself how he would feel if the figures were changed, and if, instead of our population containing one pauper in every 34, it contained one in every 6? But for medical purposes that is exactly how the matter stands, one person in every six is a pauper, about 17 in every 100, 170 in every 1,000. Every man of average sanity cannot but feel a little regard for the character of his fellow countrymen. He would not like one in every six to be a thief, or a traitor to the national flag! And yet, so far as many doctors are concerned, they willingly and eagerly reduce one in every six to the level of a pauper. One eminent doctor goes so far as to affirm that this is quite right because it has enabled him to amass an unusual amount of experimental knowledge. What! are the manly character and honest independence of a tenth of our population to be sacrificed to the professional successes of one, or half-a-dozen, or half a hundred hospital physicians? Better by half let every hospital in the kingdom from this time forward be manned as the poor law infirmaries are manned. Successful individuals, no doubt, loom very largely in their own eyes; but it is time at least for the public to give the subject a deeper consideration when millions are to be degraded to pauperism that one or a hundred may be famous and rich. If certain persons can regard with satisfaction such a turning of the world upside down as that, it is high time that other persons of larger humanity and more philosophic spirit should throw themselves into the breach. There are many ways of ruining a country. A foreign invasion may do it quickly and with great noise, but it will not do it half so effectually and permanently as will the gradual but certain pauperising of vast masses of its people.

**Disinfectants.**

The faith of medical men and the public in disinfectants is steadily growing. At the recent meeting of the British Medical Association at Leeds, it was almost unanimously agreed that the two principal means of stamping out and driving away infectious diseases of all kinds were disinfectants and isolation. The last twenty years have witnessed a diminution in all classes of zymotic diseases which has been quite remarkable. During that period the use of disinfectants has probably increased a hundredfold, whilst isolation has been carried out with a completeness and promptitude never known before. It is but fair to admit that along with these now familiar methods there has been employed much greater care in the construction, repair, and flushing of drainage. Nevertheless, to disinfectants and to isolation the greater part of the improvement in the public health is undoubtedly due. Many medical men were enthusiastically with the President of the British Medical Association, when, in eloquent language, he foreshadowed a period which would know zymotic diseases no more. That period is no doubt remote, especially in an old country like our own. But all new colonies should be able to protect themselves from zymotic scourges, and the builders of new houses should be compelled to construct drains of such simplicity as to make sewer gases almost impossible, even in our own crowded populations. The pressure of public opinion is the real force which will bring about great sanitary reformations, exactly as it has brought about other social developments. It is strange that until very recently the public took far more interest in a voyage of exploration to the north pole than they did in facts of life and death that were transpiring in their own houses. That fatal carelessness is, however, fast passing away, and every man and woman of average intelligence is now, more or less, a practical sanitarian. If to these sanitary measures the public could be persuaded to add a strong love of fresh air and exercise, and a greater simplicity of life and manners generally, there is no doubt that a still more marked improvement would take place in the happiness and health of all classes of the people.

**From Hand to Mouth.**

The story of Lachmann von Gamsenfels, who, at Stratford-on-Avon, shot the woman who loved him, her little child, and finally himself, gives melancholy emphasis to the moral we wish to draw from the terrible tragedy. Gamsenfels does not seem to have been of an unhappy disposition, nor does the woman. They had simply come to the end of their resources. From what has been stated there appears to be no doubt that he had been in receipt of a regular income of a considerable amount. But he had not put by anything for special emergencies. Presumably, he spent as he earned. Now, it is admitted that earnings may be too small to admit of "saving," and it is also undeniable that many people have no "gift" of economy. But, at the same time, it is evident to anybody capable of thinking that the present time is one that demands and insists upon stored resources. In this country there are more than sufficient hands and heads for the work that needs to be done. The risks of a "rainy day" from sickness or other cause are therefore greatly multiplied. It follows that when a man is in the sunshine of regular employment he should strive to "make hay," even if it be ever so little. The calling of a journalist, like that of a clerk, is eminently precarious. The numbers who flock into it from all sources render it unprofitable as well as precarious. The journalist who has no stored resources lives always on the edge of a volcano; it may be the volcano of hopeless poverty, or the still worse volcano of murder and suicide. Thrift is a very old-fashioned and homely virtue; but it is a prime article of faith in these struggling times with every man who has the power to look even a few months ahead. As thrift is a primary virtue, so extravagance and display are cardinal sins. Living in the midst of wealth, as so many of us do, there is a constant temptation to make as brave a show as our neighbours. The temptation is dangerous, the yielding to it may be fatal. From the point of view of health and longevity, few things are more injurious than constant anxiety about to-morrow. A balance at the bank is a daily tonic to the nervous system, of a very effectual kind. It is melancholy to think that if Gamsenfels had had but a single fifty pound note available, he and the young singer, and her child, might have been still alive and well. The pain of self-denial in order to save a little is as nothing compared with the agony of anxiety and despair which can find no relief except in murder and suicide. It is not sordid to strive to "save" when saving may mean murder averted and suicide escaped.

